# Olfactory sensitivity differentiates morphologically distinct worker castes in *Camponotus floridanus*

**DOI:** 10.1186/s12915-022-01505-x

**Published:** 2023-01-08

**Authors:** S. T. Ferguson, I. Bakis, N. D. Edwards, L. J. Zwiebel

**Affiliations:** Department of Biological Sciences, Vanderbilt University, Nashville, TN 37235 USA

**Keywords:** Hymenoptera, Olfaction, Odor coding, Task allocation, Caste, Biological Sciences - Neuroscience

## Abstract

**Background:**

*Camponotus floridanus* ant colonies are comprised of a single reproductive queen and thousands of sterile female offspring that consist of two morphologically distinct castes: smaller minors and larger majors. Minors perform most of the tasks within the colony, including brood care and food collection, whereas majors have fewer clear roles and have been hypothesized to act as a specialized solider caste associated with colony defense. The allocation of workers to these different tasks depends, in part, on the detection and processing of local information including pheromones and other chemical blends such as cuticular hydrocarbons. However, the role peripheral olfactory sensitivity plays in establishing and maintaining morphologically distinct worker castes and their associated behaviors remains largely unexplored.

**Results:**

We examined the electrophysiological responses to general odorants, cuticular extracts, and a trail pheromone in adult minor and major *C. floridanus* workers, revealing that the repertoire of social behaviors is positively correlated with olfactory sensitivity. Minors in particular display primarily excitatory responses to olfactory stimuli, whereas major workers primarily manifest suppressed, sub-solvent responses. The notable exception to this paradigm is that both minors and majors display robust, dose-dependent excitatory responses to conspecific, non-nestmate cuticular extracts. Moreover, while both minors and majors actively aggress non-nestmate foes, the larger and physiologically distinct majors display significantly enhanced capabilities to rapidly subdue and kill their adversaries.

**Conclusions:**

Our studies reveal the behavioral repertoire of minors and majors aligns with profound shifts in peripheral olfactory sensitivity and odor coding. The data reported here support the hypothesis that minors are multipotential workers with broad excitatory sensitivity, and majors are dedicated soldiers with a highly specialized olfactory system for distinguishing non-nestmate foes. Overall, we conclude that *C. floridanus* majors do indeed represent a physiologically and behaviorally specialized soldier caste in which caste-specific olfactory sensitivity plays an important role in task allocation and the regulation of social behavior in ant colonies.

**Supplementary Information:**

The online version contains supplementary material available at 10.1186/s12915-022-01505-x.

## Background

**Fig. 1:**
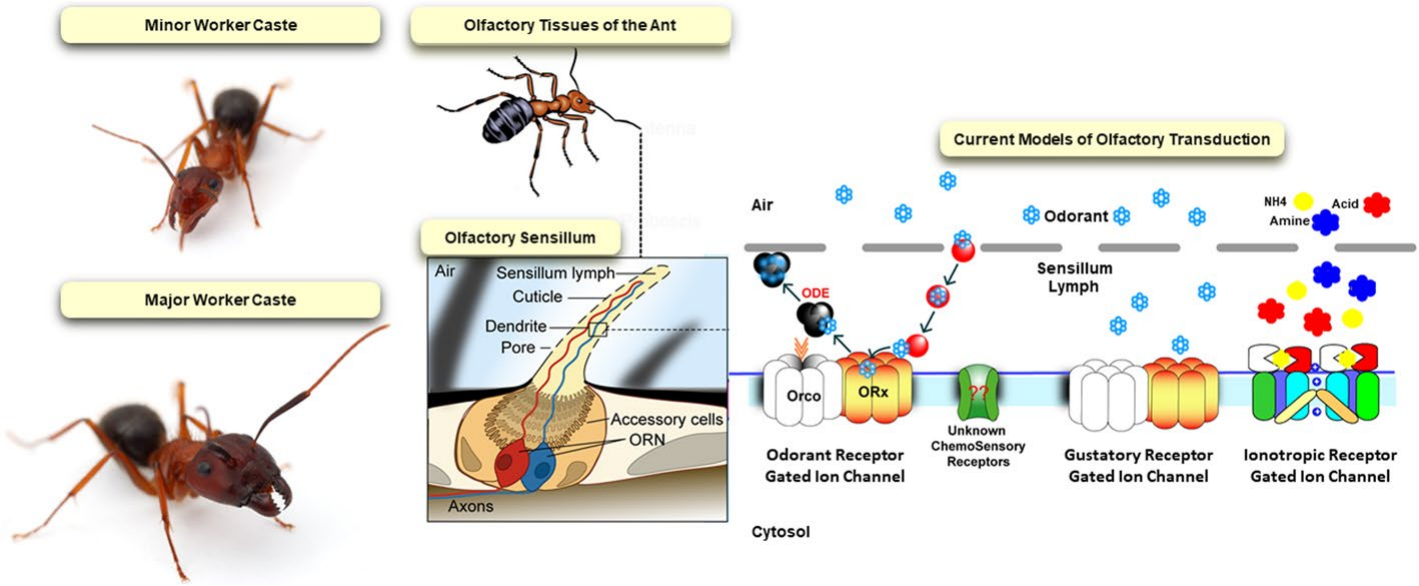
The olfactory system in *C. floridanus* minor and major workers. Minor and major workers (left) can be distinguished based on their gross morphological characteristics. Minors have a smaller body size whereas majors are larger and possess a shovel-shaped head. The initial site of olfactory signal transduction occurs in the hair-like sensory sensilla distributed across olfactory appendages such as the antennae (center) where odorants encounter one of three different chemoreceptor family members: ORs, GRs, and Irs (right)

In ants, colony survival depends on a dynamic and decentralized process of distributing work across all members of the colony [1]. Collective behaviors such as nursing offspring, foraging for food, and nest defense emerge as groups of ants detect and respond to a broad range of local information including, most notably, pheromones and other odors such as cuticular hydrocarbons (CHCs) [2]. Colonies of certain ant genera contain morphologically distinct worker castes that perform specialized roles within the colony which may reflect differences in olfactory physiology. For example, the behavioral repertoire of army ants (genus *Eciton*) is associated with worker size and shape [3, 4]. Here, the largest workers form a soldier caste that performs a restricted subset of behaviors primarily specialized for nest defense. In addition to their distinctive sharply pointed and sickle-shaped mandibles, the brain volume of these soldiers, including the antennal lobe (AL) and mushroom bodies (MB), are significantly reduced compared with other workers within the colony, suggesting that adaptive changes in these olfactory processing centers of the brain may contribute to or otherwise reflect differences in worker behavior [5]. Similarly, in the carpenter ant *Camponotus floridanus*, there are two morphological worker castes (Fig. 1) which can be readily distinguished by allometric differences in head size and shape which also follow a bimodal body size distribution within the colony [6]. Importantly, the smaller minor workers carry out the majority of tasks within the colony as well as forage outside for food and, perhaps correspondingly, have larger AL volumes and more glomeruli than their larger major worker sisters [7–9]. Transcriptome profiling in *C. floridanus* reveals that mRNA transcripts associated with muscle development are enriched in majors, whereas transcripts associated with synaptic transmission, cell-cell signaling, neurological system processes, and behavior are upregulated in the smaller, more behaviorally diverse minors [10]. Moreover, the majority of chemosensory receptors are enriched in antennal transcriptome profiles of minors compared to majors [11]. Taken together, these cellular and molecular changes raise the possibility that differences in olfactory processes also contribute to the specialized behavioral repertoire of subsets of workers.

In ants and other insects, the peripheral detection of pheromones and other chemical signals occurs principally in olfactory sensory neurons (OSNs) housed in hair-like sensilla that are distributed along the antennae and other sensory appendages (Fig. 1). OSNs express chemoreceptor gene families that include odorant receptors (ORs), ionotropic receptors (IRs) and gustatory receptors (GRs) (reviewed in [12]). Odorants enter sensilla through small pores and rapidly travel through an aqueous lymph through which they encounter these chemosensory receptors along the dendrites of OSNs. Excess odorants are cleared from the sensillar lymph by several families of odor-degrading enzymes (ODE) (Fig. 1, reviewed in [12]). The OSNs then transduce chemical information into olfactory signals as odorants induce action potentials through depolarization. These signals are relayed to the central brain via synaptic connections and processing that first begins in the AL [8, 13, 14].

Perhaps the most notable and well-studied family of chemoreceptors in ants are the ORs, which act in the detection of general odorants and critical social cues, including CHCs [15, 16]. Gene editing carried out in two primitively eusocial ants to generate null mutations of the obligate OR co-receptor (Orco) gene, which is required for the formation and functionality of odor-gated OR ion channels, resulted in significant deficits in odor sensitivity and reduced colony cohesion along with severe alterations in AL development [17, 18]. Furthermore, targeted pharmacological modulation of Orco function in wildtype *C. floridanus* workers with unaffected ALs and central brains significantly impaired aggression-mediated non-nestmate recognition [19]. Taken together, these studies highlight the critical role that olfaction plays in the detection of pheromones and other behaviorally salient odorants that ultimately act to mediate social behaviors in ants. However, considerably less is known about modulation of peripheral olfactory sensitivity to pheromones and other general odorants between morphological castes.

An appreciation as to how variation in olfactory responses contributes to or, alternatively, reflects the unique roles performed by different worker castes within the colony will inform our understanding of the emergent properties of coordinated social behavior in ants and other eusocial animals. To begin to address that question, we have undertaken a behavioral assessment as well as a broad electrophysiological survey of the antennae across approximately 400 general odorants presented as 36 distinct odor blends, non-nestmate cuticular extracts, and a known trail pheromone across the two morphological *C. floridanus* worker castes. These studies examine the hypothesis that alterations in peripheral olfactory sensitivity correlate with the distinctive social behaviors of these morphological castes. In keeping with their more diverse task engagement, peripheral responses to general odor blends and trail pheromone were significantly higher in minors than majors. Moreover, while minor workers primarily displayed a broad range of excitatory responses, majors manifested primarily sub-solvent (suppressed) responses suggesting a fundamental shift in odor coding between these castes. In contrast, CHCs and other hydrophobic compounds associated with the *C. floridanus* cuticle elicited robust excitatory responses from both majors and minors. Behaviorally, minor workers displayed a selective ability to robustly follow trail pheromone tracks while in aggression bioassays, majors were significantly more adept at rapidly subduing opponent non-nestmates with pairwise bouts often quickly resulting in maiming, dismemberment, and death. These data suggest an important role for peripheral olfactory sensitivity and odor coding in the allocation of tasks in eusocial ants and support a model in which minors are behaviorally plastic olfactory generalists and majors are a dedicated hyper-aggressive soldier caste characterized by both morphological adaptions as well as a highly-specialized olfactory system focused on the detection of CHCs and other chemical cues associated with the recognition of and defense against enemy non-nestmates.

## Results

### Peripheral electrophysiology via the electroantennogram

#### General odorants

**Fig. 2:**
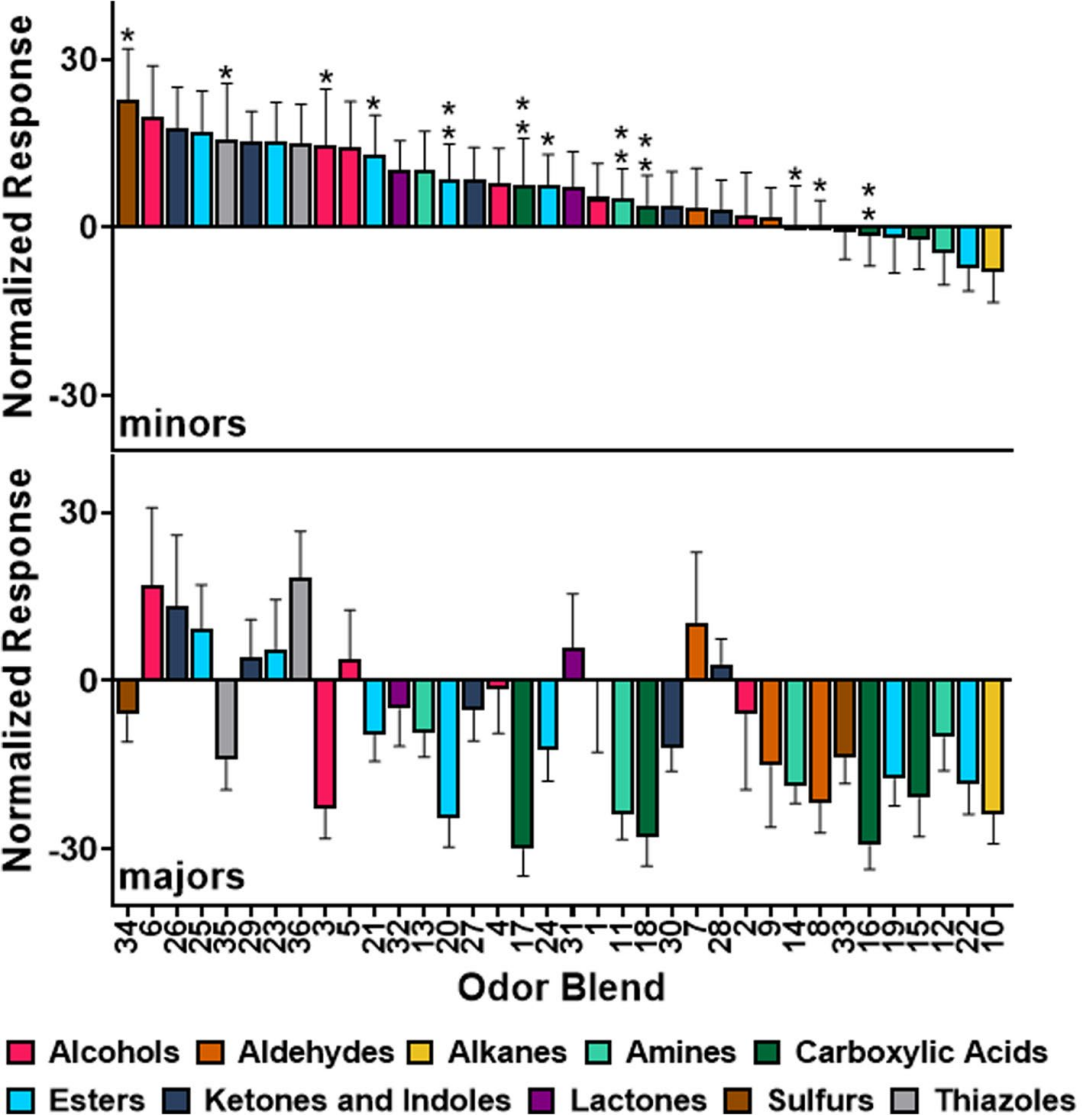
Olfactory response profiles of minor and major workers to general odorant blends. A bar graph organized by the magnitude of the solvent (ND96) normalized responses to each odor blend from high to low in minors and the corresponding responses in majors displayed below. Colors represent chemical class as indicated in the figure legend. Asterisks located above the minor bar graph indicate responses that were significantly different between minors and the corresponding response to the same blend in majors directly below (*n*=25; Welch’s *t*-test with a False Discovery Rate correction (*α*=0.05), *Q*-value: * < 0.05, ** < 0.01). Error bars represent S.E.M.

In order to facilitate the screening of a broad range of chemical stimuli, the responses of major and minor workers were examined after stimulation with a panel of 390 general odorants that were presented as 36 distinct stimulus blends organized by chemical class, including alcohols, aldehydes, alkanes, amines, carboxylic acids, esters, ketones/indoles, lactones, sulfurs, and thiazoles (Additional file 1). Such blends are commonly used to facilitate odor screening in electrophysiological experiments [20–23]. These blends collectively include a large number of odorants which evoke diverse physiological and behavioral responses across insect taxa and which may (or may not) be biologically salient for *C. floridanus*. The odorants that comprise these blends can be found in a wide range of settings and biological contexts, including floral fragrances, microbial by-products, and nutrient-derived odorants. For example, geosmin (Alcohol Blend 3) is produced by certain bacteria and fungi and has a distinctive moldy scent that elicits avoidance behavior in flies [24] but acts as an attractant for mosquitoes [25]. Benzaldhyde (Aldehyde Blend 9) can be found in almonds and other foods and is notable for its use as a bee repellent among other things [26]. Normalizing these EAG responses against solvent-alone controls not only uncovered significant differences among odor blends between minors and majors but also revealed profound odor coding distinctions (Fig. 2; Additional file 2). To begin with, the olfactory responses of minors were primarily excitatory while majors displayed a significantly higher proportion of sub-solvent responses to odor blends than minors (Fisher’s exact test, *P* < 0.0001; Additional files 2 and 3). Moreover, responses that were significantly below solvent-alone controls, perhaps suggestive of neuronal inhibition, were only observed in majors where they accounted for 17 out of the 36 blends tested (Additional file 3). These data are consistent with previous RNA sequencing studies, indicating that most olfactory gene transcripts are enriched in mature *C. floridanus* minors compared to majors (Additional file 4) [11].

A total of 12 of the 36 general odorant blends tested elicited a significantly higher response in *C. floridanus* minors than in majors (Welch’s *t*-test with a False Discovery Rate of 0.05 to correct for multiple comparisons [27], Fig. 2). Of these, 11 blends (91.7%) elicited an above-solvent response in minors and a sub-solvent response in majors, whereas one blend (8.3%) elicited a sub-solvent response in minors but nevertheless remained significantly lower in majors. Notably, these differences were consistent across different chemical classes, including alcohols (Blend 3), aldehydes (8), amines (11, 14), carboxylic acids (16–18), esters (20, 21, 24), sulfides (34), and thiazoles (35). The significantly higher responses to a broad range of chemical classes suggest these trends do not reflect a bias toward a particular set of odors but instead reveal a generalized increase in chemosensory sensitivity. Of the remaining 24 odor blends, 22 elicited higher responses in minors while only 2 provoked higher EAG responses in majors (Fig. 2).

Beyond uncovering these broadly attenuated responses in major workers, we also examined the extent to which minors and majors align with respect to the rank order of odors based on the response magnitude of each blend [28]. This hierarchical information reflects the structured relationship of one odor blend to another and may align with behavioral valence. For example, the response to Blend 6 was higher than that to Blend 26 in both minors and majors (i.e., concordance; Fig. 2). In contrast, the response to Blend 34 was higher than that to Blend 6 in minors, but the opposite was true for majors (i.e., discordance; Fig. 2). This analysis revealed that, despite profound differences in olfactory sensitivity, for any given pair of odor blends minors and majors tend to be concordant (Kendall’s rank correlation coefficient, τ = 0.384, *P* < 0.001). Among the 630 possible pairwise comparisons, 436 were concordant and 194 were discordant. These data suggest that, while response magnitude may vary significantly, the rank relationships between pairs of odor blends tend to be consistent between worker castes. The rank order of olfactory responses in minors and majors was further analyzed by examining the proportion of concordant and discordant combinations for each chemical class (Additional file 5). Here, approximately one-third of all pairwise comparisons across all chemical classes were discordant (range: 26.06–36.27%) with the exception of a singular blend of alkanes (11.43%) which elicited a relatively low response in both minors and majors (Additional file 5; Fig. 2). This suggests that there is no discernible bias or pattern amongst discordant odor pairs, but rather these trends are consistent across chemical classes. Moreover, any discordant rankings further distinguish the physiological response profiles of these morphological and behaviorally distinct castes.

#### Trail pheromone

**Fig. 3:**
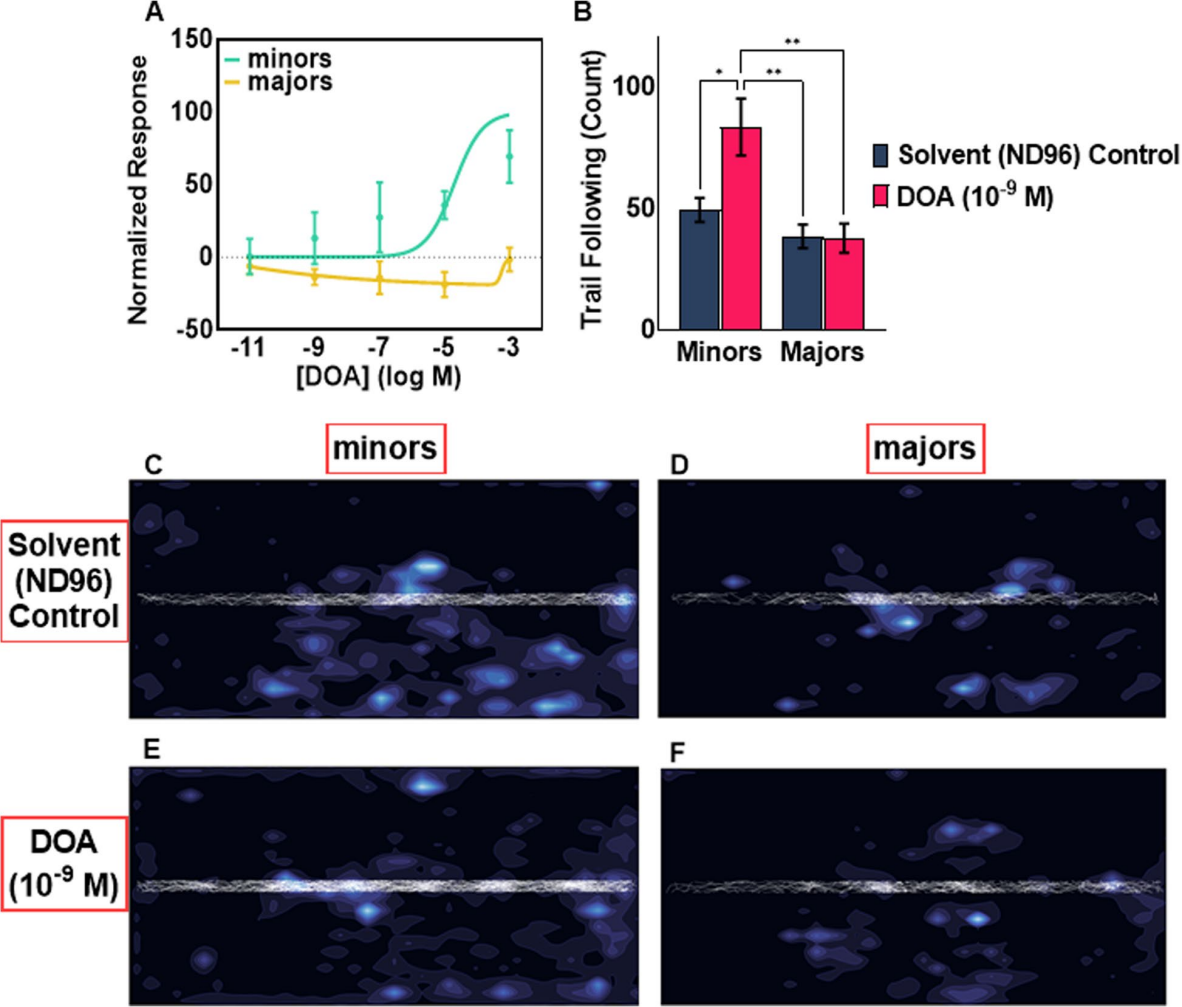
Minors but not majors display high olfactory sensitivity and trail following behaviors to DOA. **A** Solvent (ND96) normalized EAG responses showing that minors exhibited robust, dose-dependent excitatory responses to DOA whereas majors displayed hormetic responses that becomes smaller as concentration increased except at the highest concentration which saw a return to baseline (*n*=5; individual data values can be found in Additional file 9). Dots represent the mean response and error bars represent S.E.M. The lines are best fit dose response curves. **B** The mean number of instances in which minors and majors followed either the solvent (ND96) control or DOA (10^−9^ M) trail (*n*=7; two-way ANOVA with Tukey’s correction, *P*-value: * < 0.05, ** < 0.01). **C**–**F** Density contour plots pooled from the trail-following bioassays that correspond to the foraging arena with the darkest regions indicating areas of lowest density where ants were less likely to be found and the brightest regions indicating areas of max density where ants were most likely to be found. The trail (ND96 or DOA) was located along the center of the foraging arena along its length, and the white lines superimposed on the density contour plot correspond to each discrete instance in which an ant followed the trail (*n*=7)

Extending these studies beyond general odorants to pheromones and other chemical blends that are likely to be important sources of social information, we next focused on the trail pheromone nerolic acid ((2Z)-3,7-dimethylocta-2,6-dienoic acid). Previous studies have demonstrated that this compound is produced in the rectal bladder of *C. floridanus* workers and elicits a strong olfactory response and trail-following phenotype that specifically is not evoked by its double-bond structural isomer geranic acid ((2E)-3,7-dimethylocta-2,6-dienoic acid) [29]. Because purified nerolic acid is difficult and expensive to obtain, the absence of both behavioral and electrophysiological responses to geranic acid allowed us to employ a readily available isomeric mixture (Sigma-Aldrich, CAS 459-80-3) that is hereafter denoted as DOA [29]. In EAGs, minors displayed robust dose-dependent responses to DOA that generally followed a sigmoidal curve (Fig. 3A). These results are in sharp contrast to majors whose responses were slightly below solvent-alone levels at all concentrations (Fig. 3A). More specifically, the responses from majors were hormetic, i.e., becoming smaller as concentration increased except at the highest concentration tested whereby responses returned to baseline (Fig. 3A). These peripheral responses are consistent with the reduced level of foraging activity observed in majors [9]. The behavioral responses of minor and major workers to DOA were also investigated using a spotted trail-following bioassay (Additional file 6) reflecting natural foraging trails [30]. In solvent-alone controls (ND96), activity levels along the solvent trail, as measured by the mean number of times ants traversed along the length of the trail, were not significantly different between minors and majors (two-way ANOVA with Tukey’s correction, *P* = 0.7307; Fig. 3B, C, D). However, DOA elicited significantly more trail following in minors relative to solvent-alone (*P* < 0.05; Fig. 3B, C, E) whereas no significant difference in trail following was observed for majors between the solvent-alone control and DOA treatment (*P* = 0.9998; Fig. 3B, D, F). Importantly, DOA elicited significantly more trail-following behavior in minors compared to majors (*P* < 0.01; Fig. 3B, E, F). Taken together, these observations suggest that the allocation of foraging capacity to the colony’s minor workers is regulated, at least in part, by differential peripheral olfactory sensitivity to a discrete subset of salient cues that notably includes the pheromone, DOA.

#### Cuticular extracts

**Fig. 4:**
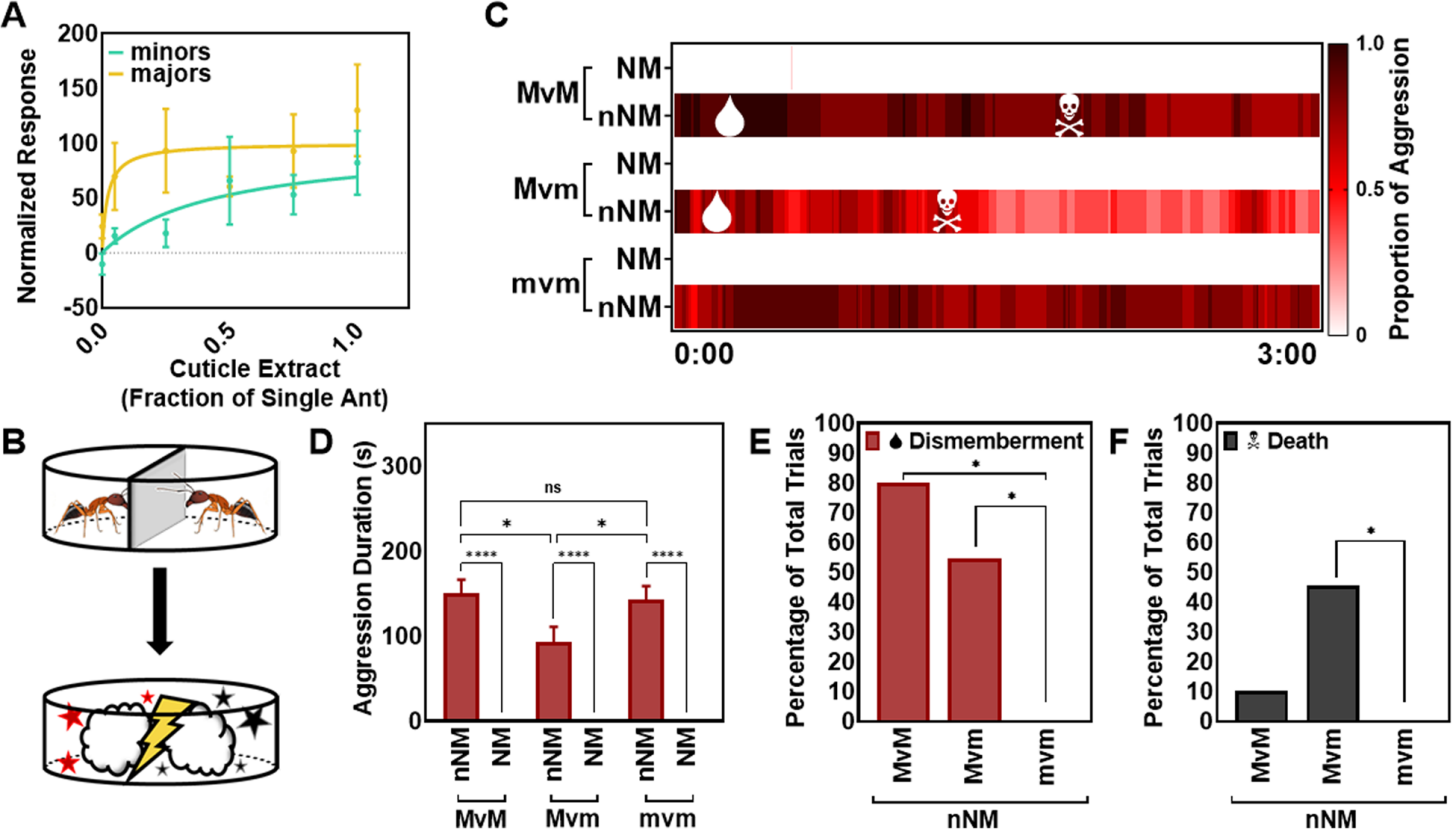
Higher olfactory sensitivity to cuticle extract in majors compared to minors aligns with more severe aggression towards non-nestmates. **A** Solvent (hexane) normalized EAG responses showing that both minors and majors exhibited robust, dose-dependent excitatory responses to cuticle extract from non-nestmate minor workers (*n*=5; individual data values can be found in Additional file 9). Dots represent the mean response and error bars represent S.E.M. The lines are best fit dose response curves. **B** Schematic of the bioassay depicting the acclimation period (top) and aggression bioassay (bottom). **C** A heat map showing the elapsed time of the 3-min aggression bioassay along the *X*-axis. The heat map represents the proportion of trials where aggression was observed at each time point for nestmates (NM) and non-nestmates (nNM) in trials conducted with majors vs. majors (MvM), majors vs. minors (Mvm), and minors vs. minors (mvm) (*n*=10–11). The mean time at which dismemberment (droplet) or death (skull and crossbones) occurred is indicated along the heat map. Notably, minor vs. minor bouts never resulted in dismemberment or death. **D** The mean aggression duration (in seconds) between nestmates and non-nestmates (*n*=10–11; two-way ANOVA with Tukey’s correction, *P*-value: ns = not significant, * < 0.05, **** < 0.0001). **E**, **F** The percentage of trials that resulted in at least one instance of dismemberment or death (*n*=10–11, *z*-test with Bonferroni’s correction, *P*-value: * < 0.016)

Previous studies in both *C. floridanus* and *C. laevigatus* have demonstrated that, although differences in the detection and perception of nestmate and non-nestmate extracts must exist, highly sensitive electrophysiological interrogation of the antennae and AL using EAGs, single-sensilla electrophysiology, or calcium imaging cannot distinguish any differences or otherwise discriminate between these responses [31, 32]. Therefore, we next compared major and minor worker responses to CHCs and other hydrophobic cuticle components by solely focusing on non-nestmate cuticle extracts, which are acutely evocative stimuli in the context of aggression [19]. Here, whole-body cuticular extracts of adult minor workers were obtained using a hexane soak at dilutions equivalent to the surface content of 0.001, 0.05, 0.25, 0.50, 0.75, or 1 individual ant. These cuticular extracts uniformly elicited robust and generally dose-dependent excitatory EAG responses from both minors and majors (Fig. 4A). While the magnitude of responses was similar in both minors and majors across the highest extract concentrations, majors generally displayed higher excitatory responses than minors. Importantly, the robust excitatory responses in majors elicited by CHC extracts contrast with the observed broadly suppressed responses to general odorant blends and trail pheromone (Figs. 2 and 3A). These provocative electrophysiological response profiles add to the distinction between *C. floridanus* minors and major worker castes.

The ability of non-nestmate cuticular extracts to elicit robust responses in majors stands in contrast to the indifference to DOA trail pheromone and the majority of other odor blends tested. This suggests that these social cues may be particularly salient to the behavioral repertoire of majors that aligns their role as a soldier caste. To further examine whether morphologically distinct major workers do indeed represent a dedicated and highly specialized soldier caste within the colony, we next quantified aggressive behaviors characteristically seen in interactions between pairs of minors and majors using a non-nestmate recognition aggression bioassay (Fig. 4B). While both minors and majors accurately accepted nestmates and, in contrast, dramatically aggressed non-nestmates (Fig. 4C), majors were adept at rapidly killing smaller non-nestmate minor worker opponents, which resulted in significantly shorter fights than in major vs. major and minor vs. minor bouts (Tukey’s comparison, *P* < 0.0001; Fig. 4D). Moreover, majors were responsible for all acts of severe and lethal aggression and were significantly more likely to dismember and kill opponent non-nesmates (*z*-test with Bonferroni’s correction, *P* < 0.016). Taken together with the electrophysiology, these behavioral distinctions suggest that non-nestmate recognition signals present on the cuticle are detected, perceived, and acted upon by both majors and minors. However, majors respond more robustly, are more aggressive, and therefore represent significantly superior fighters that are capable of rapidly killing their opponents.

## Discussion

In eusocial ant colonies, behavioral patterns such as nursing, foraging, and colony defense emerge from the collective behavior of workers. These emergent social behaviors occur through a decentralized distribution of work across members of the colony known as task allocation [1], which is the outcome of both intrinsic and extrinsic factors [1, 33–37]. While behavioral caste is often associated with age [38–40], ant workers also switch tasks in response to a wide range of chemical and tactile signals that convey situational changes in colony requirements [33, 41]. These peripheral stimuli are integrated and decoded in various parts of the ant brain, which has a remarkable capacity to discriminate subtle features of information [42, 43]. Here, we have extended these studies through a large electrophysiological screen of general odorants, pheromones, and cuticular odor blends complemented by a series of behavior bioassays to test the hypothesis that variation in peripheral olfactory sensitivity of *C. floridanus* major and minor workers correlates with caste-specific differences in their behavioral repertoire.

Previous studies have demonstrated that in *C. floridanus* [9] and other ant species [44], minor workers performed the majority of tasks within the colony, such as tending to the brood and foraging for food. By contrast, *C. floridanus* majors rarely forage and seldom, if ever, tend to the brood. This apparent inactivity explains why the precise role of *C. floridanus* majors has remained largely enigmatic. To begin to address this question, we examined the hypothesis that the behavioral repertoire of workers aligns with distinctive olfactory profiles. In doing so we uncovered profound differences in peripheral olfactory sensitivity and odor coding between *C. floridanus* minors and majors. To begin with, minor workers were significantly more sensitive to general odorants (Fig. 2) and trail pheromone (Fig. 3A). These observations aligned with the ability of minors, but not majors, to robustly detect and follow DOA as a trail pheromone (Fig. 3). Furthermore, general odorant stimuli elicited primarily excitatory responses from minors whereas majors displayed primarily sub-solvent responses (Fig. 2, Additional file 2). Although the extent to which sensilla and neuronal density or other morphological differences might influence the olfactory responses of minors and majors remains unclear, we posit that the sub-solvent olfactory responses displayed by majors should not simply be considered anosmic, i.e., null (= 0) responses comparable to the solvent-alone background controls. Instead, we interpret these large, sub-solvent responses (Fig. 2, Additional file 2) as *bona fide* inhibitory responses and, in that context, biologically salient signals in their own right [45].

That the expanded repertoire and higher engagement in social behavior by minor workers is associated with broad excitation and detection of general odorants and pheromones and contrasts with low activity and diminished responsiveness in majors provides an intriguing link between the regulation of peripheral olfactory physiology and task allocation. While we are currently unable to fully appreciate the odor coding implications of excitatory versus inhibitory EAG responses, these data align with models of task allocation that propose behavioral performance depends on internal differences between individuals with respect to stimulus thresholds [46]. In these models, when a particular task-related stimuli exceeds an internal threshold, the worker will engage in the task. While response thresholds are likely not the only determinant of behavior [47], they may nevertheless contribute to the overall division of labor in social insect colonies [48, 49]. Our data suggest that differences in the peripheral detection and odor coding of chemical stimuli by the antennae represent the initial mechanism through which these thresholds manifest.

Interestingly, in contrast to general odorants and trail pheromone, both minors and majors displayed robust, dose-dependent excitatory responses to non-nestmate cuticular extracts (Fig. 4A). Importantly, the olfactory responses of majors to these stimuli were higher than those of minors (Fig. 4A). If task performance depends on physiological stimulus thresholds, then it stands to reason that *C. floridanus* majors are likely to be involved in robust aggressive behaviors towards non-nestmates to the exclusion of other tasks. Consistent with this hypothesis, we found that while both minors and majors could accurately discriminate between nestmates and non-nestmates, major workers were significantly more effective at rapidly dismembering and killing non-nestmates during aggressive interactions (Fig. 4C–E). This distinctive electrophysiology and aggressive behavior suggest that while non-nestmate recognition signals are detected and acted on by both castes, majors are categorically and quantitatively more effective fighters. This reflects not only the superior size and mandibular characteristics of majors, but also and importantly, their enhanced and seemingly highly specialized chemosensory responses to discrete non-nestmate stimuli that appear to manifest at the exclusion of most other responses.

These electrophysiological and behavioral distinctions are consistent with similar observations made in other ant species. For example, the larger major workers of the leaf-cutting ant *Acromyrmex echinatior* are significantly more aggressive to conspecific non-nestmates than the smaller minor workers, even if they possess what appears to be a relatively similar cuticular profile [50]. In this light, the high levels of aggression in *C. floridanus* majors we observe are not surprising. However, when viewed together with our detailed peripheral electrophysiological interrogation of the antennae, these results support the hypothesis that behavioral distinctions in ants may initially arise, at least in part, due to profound differences in peripheral olfactory sensitivity and odor coding between majors and minors. Indeed, the peripheral olfactory system of majors appears to be selectively specialized for the detection of CHCs and other salient compounds specifically linked to a narrow range of social behaviors that require the recognition and aggressive rejection of non-nestmates as potential threats to colony integrity. These data align with previous observations in *Atta mexicana* demonstrating that responses to oleic acid and flower essence, but not 2-heptanone, are lower in soldiers relative to foragers [51]. These findings also contribute to a growing body of evidence that aggression in ants is associated with distinctive levels of brain neuromodulators, most notably octopamine and serotonin [52, 53]. Here, we demonstrate these critical variations also exist in the odor coding apparatus of the antennae which, from a signal transduction perspective, lies upstream of the brain and are indeed the initial steps in the process of olfactory detection that elicits downstream perception and behavioral responses. We posit that these differences in peripheral olfactory sensitivity are evolutionary adaptations that reflect the unique physiology and behavior of ant species which have evolved specialized morphological caste. Whether such variation exists within monomorphic ant species and how such variation contributes to behavioral differences within castes remains an intriguing question for future research.

## Conclusions

Our data are consistent with the hypothesis that minors are multipotential jacks-of-all trades, engaged in a diverse set of social behaviors in the colony with broad, excitatory olfactory sensitivity to general odorants and pheromones. Majors, however, are a highly specialized soldier caste dedicated to colony defense, the olfactory system of which is fine tuned to detect non-nestmate chemical signatures. Taken together, these results suggest that directed shifts in olfactory sensitivity play important roles in establishing and maintaining caste identity as well as the allocation of social behaviors within a eusocial collective.

## Methods

### Animal husbandry

Ants for these studies were taken from one of six laboratory-reared colonies of *Camponotus floridanus* (Buckley 1866) originating from field collections from the Sugarloaf Key (D601) and the Fiesta Key (C6, K17, K19, K34, and K39) in South Florida, USA, that were generously gifted by the laboratories of Dr. J. Liebig (Arizona State University) and Dr. S. Berger (University of Pennsylvania), respectively. The colonies were separately maintained at 25°C with an ambient humidity of approximately 70%. Colonies were stored in an incubation chamber with a 12 h light:12 h dark photoperiod. Each colony was provided with Bhatkar diet, crickets, 10% sucrose solution, and distilled water three times per week. Adult minor and major workers were used for all experiments.

### Olfactory stimuli: unitary odorants, odor blends, and cuticular hydrocarbons

Compounds of the highest purity, typically ≥99% (Sigma-Aldrich), were used to make v/v (for liquids) or m/v (for solids) solutions at specified concentrations. One-molar (1-M) stocks of each compound were prepared in DMSO and subsequently diluted in ND96 (96 mM NaCl, 2 mM KCl, 1 mM MgCl2, 1 mM CaCl2, and 5 mM HEPES, pH 7.5). A total of 390 unitary compounds were used to create 36 blends each of which comprised 9–17 compounds of the same functional class at 1×10^−3^ M each (Additional file 1). Cuticular extracts were obtained by soaking 40 minor nestmates in 8 ml hexane, a non-polar solvent useful for extracting hydrophobic cuticular compounds in ants, for 30 min. This cuticle soak was then decanted, and the hexane was evaporated using compressed nitrogen. The remaining contents of the extraction were then resuspended in hexane so that odor cartridges were filled with 20 μl hexane or hexane plus extract at the appropriate concentration (equivalent to cuticle soak obtained from 0.001, 0.05, 0.25, 0.50, 0.75, or 1 whole ant). Given that nerolic acid ((2Z)-3,7-dimethylocta-2,6-dienoic acid) is detected and elicits robust trail following in *C. floridanus* workers but its isomer geranic acid ((2E)-3,7-dimethylocta-2,6-dienoic acid) is neither detected nor followed [29], nerolic acid was obtained as a mixture of isomers (Sigma-Aldrich, CAS 459-80-3) comprised of roughly 70% trans-geranic acid and about 20–25% nerolic acid (personal communication with manufacturer) which we refer to as simply DOA. DOA was then serially diluted in ND96 to concentrations of approximately 10^−11^, 10^−9^, 10^−7^, 10^−5^, and 10^−3^ M with respect to the nerolic acid isomer.

### Electroantennography

Electroantennograms (EAGs) were conducted using an IDAC-232 controller (Ockenfels Syntech GmbH, Buchenbach, Germany) and data were initially collected and stored on EAG2000 software (Ockenfels Syntech GmbH). Odorant volatiles were delivered as the headspace above filter-paper discs (VWR, Whatman VWR, West Chester PA) onto which 10 μl of each odor (or solvent for control) was aliquoted before being inserted inside glass Pasteur pipette odorant cartridges that can be inserted into a constantly maintained stimulus delivery air flow of 1.5 cm^3^ s^−1^ the output of which is placed proximate to the sample preparation [54]. Volatiles were delivered as 500 ms stimulus pulses using a Syntech CS-05 stimulus flow controller (Ockenfels Syntech GmbH). In order to increase the signal-to-noise of our *C. floridanus* EAG recordings, these electrophysiological setups were modified to significantly restrict worker movement. Minors were placed in a 20-μl disposable pipette tip that was modified such that the tip opening was sufficiently wide to allow the unimpeded exposure of the head and antennae. Majors were placed in modified 200-μl pipette tips to accommodate their wider head. To further prevent unwanted movement from the ant that might otherwise interfere with the quality of the recording, the head, mandibles, and right (ventral) antennae of the ant were tightly constrained with wax. Borosilicate glass capillaries (FIL, o.d. 1.0 mm, World Precision Instruments, Inc.) were prepared using a P-2000 laser micro-pipette puller (Sutter Instruments). Both the reference electrode and the recording electrode were backfilled with 10^−1^ mol l^−1^ KCl and 0.05% PVP buffer and placed over tungsten electrodes. Due to the armor-like exoskeleton of the ant, a 30-gauge needle was required to puncture the right eye prior to inserting the reference electrode. The recording electrode was placed over the distal tip of the left antenna. Odorants were only delivered when the amplitude of the EAG signal returned to baseline (0 mV) in approximately one-minute intervals. The resulting signals were amplified 10× and imported into a Windows PC via an intelligent data acquisition controller (IDAC, Syntech, Hilversum, The Netherlands) interface box. These recordings were analyzed using EAG software (EAG Version 2.7, Syntech, Hilversum, The Netherlands) such that stimulus-evoked response amplitudes were normalized using linear interpolation and then subtracting the response amplitude of control (solvent alone) responses.

The heterocyclic compound 5,6,7,8-tetrahydroquinoline (TETQ) was diluted in diethyl ether to 10^−1^ M for use as a positive control to ensure the integrity of the biological preparation for each EAG experimental setup. Prior to all experimental EAG recordings, the preparation was first stimulated with the headspace of 10 μl diethyl ether, then TETQ (10^−1^ M), and then another control stimulus of diethyl ether alone. Normalization of the TETQ response was subsequently done through linear interpolation vis-à-vis EAG2000. If the TETQ response was greater than 1.5× solvent alone, then the experimental preparation was deemed valid and further recordings would commence. If the TETQ response was less than 1.5× solvent the experimental setup was considered defective (resulting from a variety of possible causes that include both biological and technical issues) and subsequent recordings were not conducted. Following a previously published protocol initially used for two-electrode voltage clamp electrophysiology but employed here for EAGs [23], odor blends were created based on chemical class and then diluted in ND96 buffer (96 mM NaCl, 2 mM KCl, 1 mM MgCl2, 1 mM CaCl2, and 5 mM HEPES, pH 7.5) with each individual odorant diluted to a final concentration of 10^−3^ M. Odor cartridges were filled with 10 μl of solution. Recordings were conducted using the full panel of odor blends in the following order: ND96, odor blends 1-18, ND96, odor blends 19-36, ND96 (*n*=25 minors; 25 majors). In this way, responses could be normalized to solvent responses recorded across the duration of the trial to account for antennae degradation over time throughout the assay.

When testing responses to hexane alone or cuticular extract, a handheld butane torch (BernzOmatic, Worthington Industries, Columbus, OH, USA) was used to volatilize the cuticular compounds by heating the odor cartridge for 1.5 s. Odors were introduced in the following order: hexane, cuticle extract, hexane (*n*=5 minors; 5 majors). When testing DOA, odors were then introduced in the following order: ND96, DOA, ND96 (*n*=5 minors; 5 majors) so that, as before, responses could be normalized to solvent responses recorded across the duration of the trial to account for antennae degradation over time throughout the assay.

### Trail-following bioassay

Foraging arenas were constructed using a 21.59×27.94 cm sheet of grade 703 blotting paper (VWR International™) with 12 μl of either solvent (ND96) or DOA (as a mixture of isomers) distributed evenly as six 2-μl droplets every 4 cm along the length of the center of the paper using a pipette (Additional file 6). For each replicate (*n*=7), ten adult minor or major workers were removed from a randomly selected colony and placed in a Petri dish to acclimate for at least 5 min. After allowing the solvent to evaporate for 5 min, the ants were introduced into the arena and their activity was subsequently digitally recorded for 5 min. To assist with the identification and tracking of individual ants in the digital video recordings of each foraging bioassay, videos were first cropped using Adobe Premiere Pro to include only the foraging arena of the bioassay, and the brightness and contrast were manually adjusted so that the background appeared white and the ants appeared black. Animal tracking was performed in Python using a modified centroid tracking algorithm detailed in [55] (Additional files 7 and 8). To quantify the level of trail-following activity, we counted the number of instances ants walked along a horizontal path that fell within 0.5cm of the center of the paper which would align with the location of the ND96 solvent or DOA trail (Additional file 6).

### Aggressive recognition of non-nestmates

Aggression bioassays were modeled after a previously published method (see [19]). Briefly, nestmate (*n*=10) and non-nestmate (*n*=10 minor vs. minor; *n*=10 major vs. major; *n*=11 minor vs. major) workers were randomly assigned to the following treatment groups: minor vs. minor, minor vs. major, and major vs. major. Two workers were placed in each half of an individual well in a six-well culture plate with a plastic divider separating the ants in the middle. The ants were allowed to acclimate for 13 min before removing the divider and recording their interactions for 3 min using a digital, high-definition camera (Panasonic® HC-V750) at 30 frames per second. Videos were then scored by a blinded observer. Each frame of the video received a binary score of either 0 (no aggression) or 1 (aggression) depending on the behavior of the ants at each time point. To calculate total duration of aggression, the number of frames in which aggression took place was divided by the total number of frames (5401) throughout the trial, and then converted to s given that 1 frame was equal to 1/30 s. Aggression behavior included mandible opening, lunging, biting, spraying formic acid, grappling, and dismemberment.

### Statistical analyses

Prism 9.1.2 (Graphpad Software ©) was used to create most graphics figures and for most statistical testing including (1) Welch’s *t*-tests with a False Discovery Rate of 0.05 to correct for multiple comparisons using the two-stage step-up method described in [27] (Fig. 2); (2) non-linear regression (Figs. 3A and 4A); (3) two-way ANOVAs with Tukey’s correction (Figs. 3B and 4D); and (4) Fisher’s exact test (Additional file 3). Prism was also used to conduct a series of One-Sample t-tests; however, MultiPy [56] was used to correct for multiple comparisons [27] as Prism lacks this function when conducting a series of one-sample *t*-tests (Additional file 3). SciPy’s [57] statistical function stats.kendalltau was used to calculate the τ statistic and *P*-value with respect to Kendall’s rank correlation coefficient (Tau-b, τ). To visualize discordant pairs, we used MNE-Python [58] to create chord diagrams (Additional file 5). *Z*-tests were performed using the calculator provided in [59] and Bonferroni’s correction determined manually (Fig. 4E, F). Animal tracking was performed in Python (3.9.12) using a modified centroid tracking algorithm detailed in [55] (Additional files 7 and 8 and publicly available for download on Figshare repository at doi.org/10.6084/m9.figshare.21672965) and graphs were created using Plotly (Fig. 3C–F). The following Python libraries and their respective versions were used: SciPy (1.7.3), Numpy (1.21.5), argparse (1.1), Pandas (1.4.2), imutils (0.5.4), and OpenCV (4.6.0). Raw data presented in this manuscript can be found in Additional file 9. Animal tracking data has been made publicly available for download on Figshare repository at doi.org/10.6084/m9.figshare.21672644.

## Supplementary Information

**Additional file 1.** General odorants. A list of the general odorant blends tested.

**Additional file 2.** Representative traces of excitatory responses in minors and inhibitory responses in majors. Representative EAG traces from six different odor blends: Alcohol Blend 3 (red), Amine Blend 13 (light green), Carboxylic Acid Blend 18 (dark green), Ester Blend 20 (blue), Sulfur Blend 34 (brown), and Thiazole Blend 35 (gray) so minors (left) and majors (right) with the average ND96 solvent response across the recording displayed by the dashed gray line.

**Additional file 3.** General odorants elicit significantly different responses in minors and majors. Pie chart showing the proportion of general odor blends (total=36) that were significantly above solvent (i.e. significantly above a normalized EAG response of 0) (dark red), above solvent but not significantly different (ns) (light red), below solvent but not significantly different (light blue), and significantly below solvent (dark blue) for minor and major workers (One-Sample t-tests with a False Discovery Rate correction (α=0.05)). Majors displayed a significantly higher proportion of sub-solvent responses to odor blends than minors (Fisher’s Exact Test, P < 0.0001).

**Additional file 4 **Chemoreceptor transcripts are enriched in minor workers compared with majors. The antennae of minor *C. floridanus* workers are enriched for the three primary classes of chemoreceptors: ORs, GRs, and IRs. Figure derived from data published in [11]. Red fill indicates genes that were significantly enriched in either caste. Points that fall to the left or right of the dotted lines indicate genes that were not detected in minors and majors, respectively.

**Additional file 5.** Discordant odorant pairs organized by chemical class. Chord diagrams for each chemical class representing discordant pairwise comparisons between minors and majors according to Kendall’s rank correlation test. All other possible pairwise comparisons not drawn in the chord diagram are concordant. The colors in each chord diagram correspond to a single odor blend within the given chemical class.

**Additional file 6.** Spotted trail following bioassay. Ten nestmate minor or major workers were placed in a foraging arena with six 2 μl droplets of either ND96 solvent or 10^-9^ M DOA evenly distributed every 4 cm across the center of the arena (shown as red dots) (left). Trials were digitally recorded, and a computer vision program was used to identify and track the movement of individual ants across the arena (center). Paths that traversed the along the length of the trail were recorded as trail following events (shown as red dotted lines) whereas all other movement including movement perpendicular to the trail were not counted as trail following events (shown as blue dotted lines) (center). Density contour plots were then created and superimposed with the trail following events (shown as solid white lines) (right).

**Additional file 7.** CentroidTracker. Python script which contains the CentroidTracker class.

**Additional file 8.** Object-Oriented Tracker. Python script which contains the object-oriented animal tracker.

**Additional file 9.** Raw data.

## Data Availability

All data generated or analyzed during this study are included in this published article and its supplementary information files. The Python code used to perform the object-oriented animal tracking (Fig. 3B–F) is available in Additional files 7 and 8 and has been made publicly available for download on Figshare repository at doi.org/10.6084/m9.figshare.21672965. The raw animal tracking data (Fig. 3B–F) has been made publicly available for download on Figshare repository at doi.org/10.6084/m9.figshare.21672644. All other raw data has been made available in Additional file 9.

## Abbreviations

CHCs: Cuticular hydrocarbons
AL: Antennal lobe
MB: Mushroom bodies
LH: Lateral horn
OSNs: Olfactory sensory neurons
ORs: Odorant receptors
GRs: Gustatory receptors
IRs: Ionotropic receptors
Orco: OR co-receptor
DOA: 3,7-Dimethyl-octadienoic acid
TETQ: 5,6,7,8-tetrahydroquinoline
EAGs: Electroantennograms
MvM: Major vs. major
Mvm: Major vs. minor
mvm: Minor vs. minor
NM: Nestmate
nNM: Non-nestmate
S.E.M.: Standard error the mean
ZT: Zeitberger time
