# Supplementary figures and images for: Olfactory sensitivity differentiates morphologically distinct worker castes in Camponotus floridanus
Source: BMC Biol. 2023 Jan 8;21:3. doi: 10.1186/s12915-022-01505-x (PMC9827628; doi:10.1186/s12915-022-01505-x)

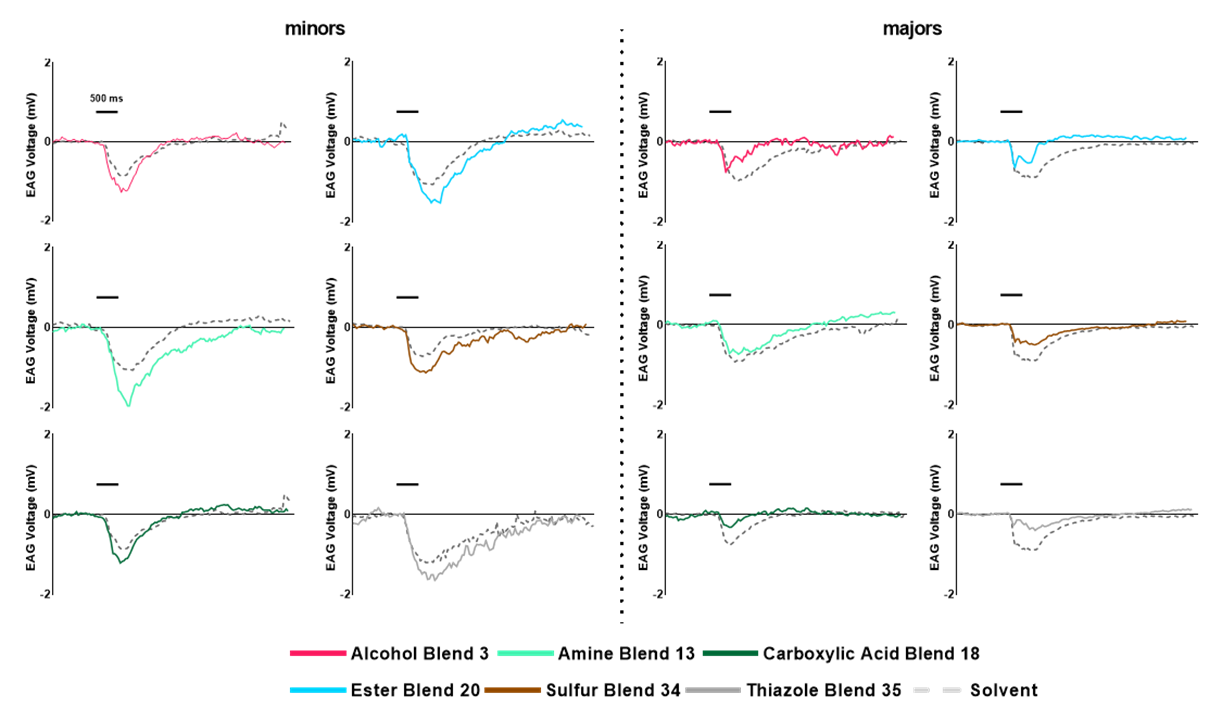

Supplement: Supplementary file 2 — Additional file 2. Representative traces of excitatory responses in minors and inhibitory responses in majors. Representative EAG traces from six different odor blends: Alcohol Blend 3 (red), Amine Blend 13 (light green), Carboxylic Acid Blend 18 (dark green), Ester Blend 20 (blue), Sulfur Blend 34 (brown), and Thiazole Blend 35 (gray) so minors (left) and majors (right) with the average ND96 solvent response across the recording displayed by the dashed gray line. [file 12915_2022_1505_MOESM2_ESM.tif]

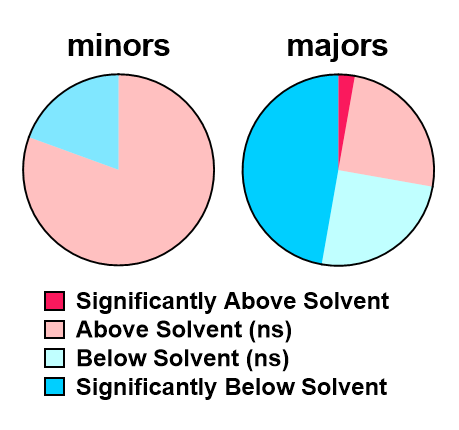

Supplement: Supplementary file 3 — Additional file 3. General odorants elicit significantly different responses in minors and majors. Pie chart showing the proportion of general odor blends (total=36) that were significantly above solvent (i.e. significantly above a normalized EAG response of 0) (dark red), above solvent but not significantly different (ns) (light red), below solvent but not significantly different (light blue), and significantly below solvent (dark blue) for minor and major workers (One-Sample t-tests with a False Discovery Rate correction (α=0.05)). Majors displayed a significantly higher proportion of sub-solvent responses to odor blends than minors (Fisher’s Exact Test, P < 0.0001). [file 12915_2022_1505_MOESM3_ESM.tif]

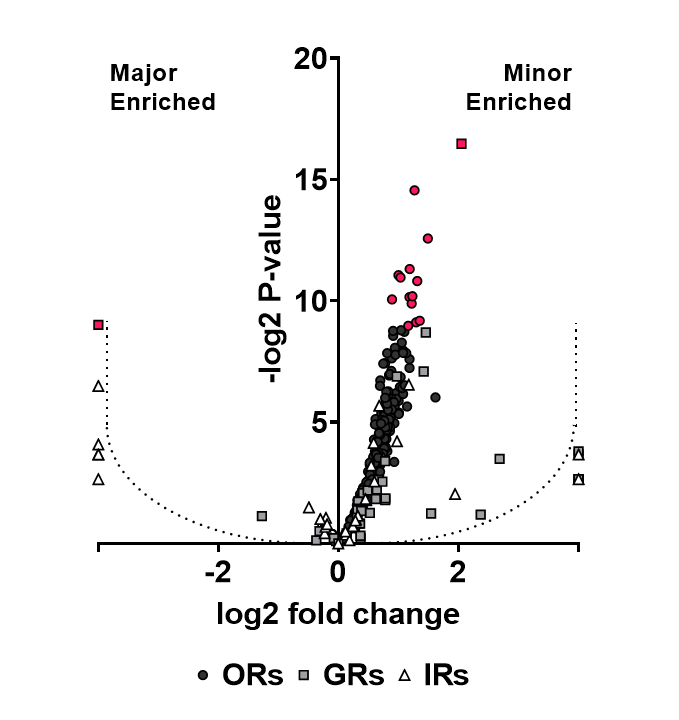

Supplement: Supplementary file 4 — Additional file 4 Chemoreceptor transcripts are enriched in minor workers compared with majors. The antennae of minor C. floridanus workers are enriched for the three primary classes of chemoreceptors: ORs, GRs, and IRs. Figure derived from data published in [11]. Red fill indicates genes that were significantly enriched in either caste. Points that fall to the left or right of the dotted lines indicate genes that were not detected in minors and majors, respectively. [file 12915_2022_1505_MOESM4_ESM.tif]

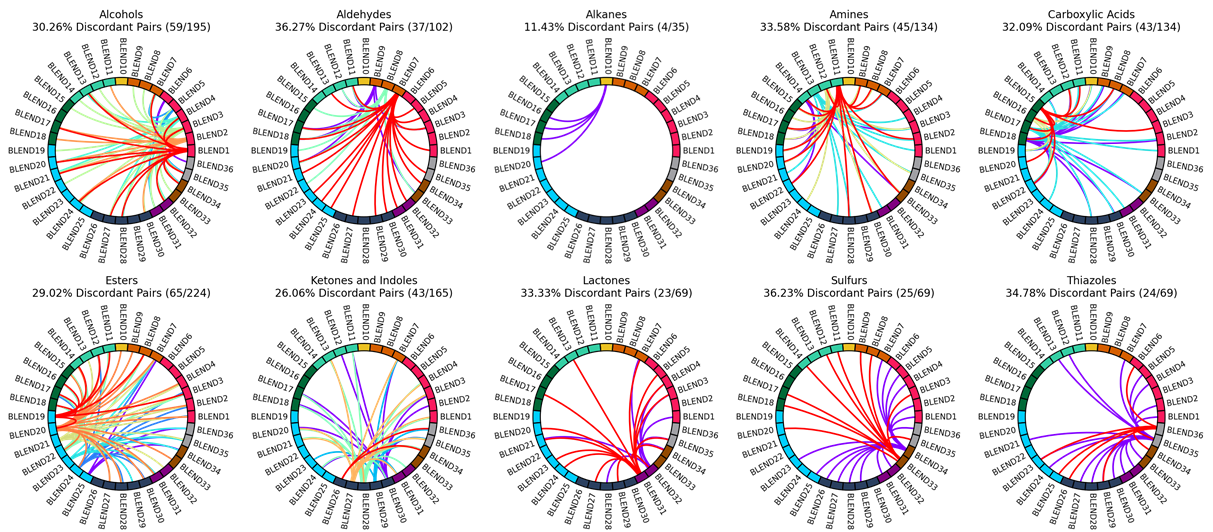

Supplement: Supplementary file 5 — Additional file 5. Discordant odorant pairs organized by chemical class. Chord diagrams for each chemical class representing discordant pairwise comparisons between minors and majors according to Kendall’s rank correlation test. All other possible pairwise comparisons not drawn in the chord diagram are concordant. The colors in each chord diagram correspond to a single odor blend within the given chemical class. [file 12915_2022_1505_MOESM5_ESM.tif]

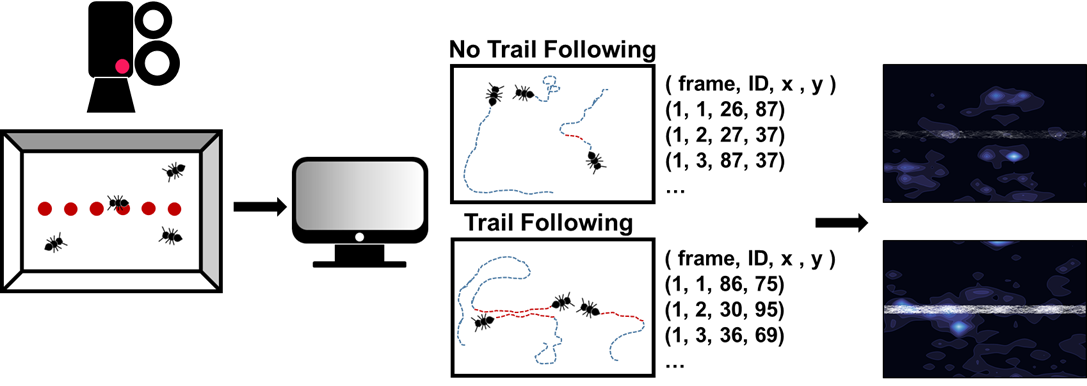

Supplement: Supplementary file 6 — Additional file 6. Spotted trail following bioassay. Ten nestmate minor or major workers were placed in a foraging arena with six 2 μl droplets of either ND96 solvent or 10-9 M DOA evenly distributed every 4 cm across the center of the arena (shown as red dots) (left). Trials were digitally recorded, and a computer vision program was used to identify and track the movement of individual ants across the arena (center). Paths that traversed the along the length of the trail were recorded as trail following events (shown as red dotted lines) whereas all other movement including movement perpendicular to the trail were not counted as trail following events (shown as blue dotted lines) (center). Density contour plots were then created and superimposed with the trail following events (shown as solid white lines) (right). [file 12915_2022_1505_MOESM6_ESM.tif]
